# Preparation, characterisation and biological evaluation of biopolymer-coated multi-walled carbon nanotubes for sustained-delivery of silibinin

**DOI:** 10.1038/s41598-020-73963-8

**Published:** 2020-10-09

**Authors:** Julia Meihua Tan, Saifullah Bullo, Sharida Fakurazi, Mohd Zobir Hussein

**Affiliations:** 1 Materials Synthesis and Characterisation Laboratory, Institute of Advanced Technology (ITMA), Universiti Putra Malaysia, Serdang, 43400 Selangor, Malaysia; 2 Department of Human Anatomy, Faculty of Medicine and Health Sciences, Universiti Putra Malaysia, Serdang, 43400 Selangor, Malaysia

**Keywords:** Drug delivery, Medicinal chemistry, Toxicology

## Abstract

This research work represents the first major step towards constructing an effective therapeutic silibinin (SB) in cancer treatment using oxidised multi-walled carbon nanotubes (MWCNT-COOH) functionalised with biocompatible polymers as the potential drug carrier. In an attempt to increase the solubility and dispersibility of SB-loaded nanotubes (MWSB), four water-soluble polymers were adopted in the preparation process, namely polysorbate 20 (T20), polysorbate 80 (T80), polyethylene glycol (PEG) and chitosan (CHI). From the geometry point of view, the hydrophobic regions of the nanotubes were loaded with water-insoluble SB while the hydrophilic polymers functionalised on the outer surfaces of the nanotubes serve as a protective shell to the external environment. The chemical interaction between MWSB nanocomposites and polymer molecules was confirmed by Fourier transform infrared spectroscopy (FTIR) and Raman spectroscopy. Besides, high-resolution transmission electron microscopy (HR-TEM), field emission scanning electron microscopy (FESEM), thermogravimetric analysis (TGA) and UV–visible spectrophotometry were also employed to characterise the synthesised nanocomposites. The morphological study indicated that the polymers were deposited on the external surfaces of MWSB and the nanocomposites were seen to preserve their tubular structures even after the coating process was applied. The TGA results revealed that the incorporation of biopolymers practically improved the overall thermal stability of the coated MWSB nanocomposites. Evaluation of the in vitro effect on drug release rate by the nanocomposites was found to follow a biphasic release manner, showing a fast release at an initial stage and then a sustained-release over 2500 min. Besides, the drug release mechanisms of the nanocomposites demonstrated that the amount of SB released in the simulated environment was governed by pseudo-second order in which, the rate-limiting step mainly depends on diffusion of drug through chemisorption reaction. Finally, MTT assay showed that the coated MWSB nanocomposites on 3T3 cells were very much biocompatible at a concentration up to 100 g/mL, which is an evidence of MWSB reduced cytotoxicity.

## Introduction

Carbon nanotubes (CNT), formed from graphene sheets rolled up into seamless cylindrical tubes with a diameter of the order of one nanometre, are a relatively new carbon allotrope since its first discovery in 1991 by a Japanese scientist called Sumio Iijima^1^. This promising nanomaterial is classified as single-walled carbon nanotubes (SWCNT) and multi-walled carbon nanotubes (MWCNT), depending on the number of sidewalls present. Over the last few decades, the exploitation of CNT and its functionalised derivatives are the focus of intense interest worldwide due to their peculiar geometrical structure (greater surface area) and excellent characteristics (electrical, chemical, thermal and mechanical properties) for diverse multifunctional applications^2–4^. With advances in nanotechnology that serve as the scientific beacon of the future, many reported systems of CNT are being extensively studied for their immense potential especially in therapeutic and diagnostic applications like site-specific targeted drug delivery, biosensing and bone regeneration treatment^5–9^.

In the field of biomedical applications, CNT displays numerous advantages compared with other drug delivery platforms. The internal cavities of these versatile nanotubes can provide larger drug loading capacity for many therapeutic agents including anticancer drugs, proteins and nucleic acids^10–12^. Owing to the CNT’s unique architecture, a wide range of bioactive molecules can also be immobilised (covalently or non-covalently) on the external surface of CNT for specific targeting purposes^13–17^. Furthermore, the tubular shape of CNT at the minuscule level enables them to better penetrate cell membranes like a needle via endocytosis without causing morphological characteristic cell changes and death^18^.

However, expanded use of CNT in biological systems suffered from various toxicology profiles due to their poor dispersion in aqueous medium caused by agglomeration and are therefore deemed unsuitable for pharmacological use^19,20^. In addition, some research groups have reported that the toxicological effects of carbon-based nanomaterials might be related to other factors such as the presence of metallic catalyst residues in their growth process ^21,22^ as well as the CNT’s purity and structure^23^ that could lead to heart problem and blood clot^24,25^. Even so, scientists have yet to determine the precise reason for their potential human health effects in biological conditions owing to a lack of thorough study on the toxicological assessments. To overcome the biological barriers of the said cytotoxicity effects posed by the nanotubes, various research projects related to surface functionalisation using biocompatible polymers through chemical bonding or wrapping methods have been exclusively reported in the literature^26–28^. From a chemical reactivity point of view, these efforts are mainly dedicated to enhance the solution processability and biocompatibility of the system in vivo and in vitro, which will expand the pharmaceutical applications of CNT as an excellent drug delivery vehicle.

With regard to drug delivery in nanomedicine, the blending of CNT with a natural biocompatible polymer or water-soluble surfactant is a very effective technique to circumvent this obstacle and it has proven distinct advantages for long-term sustained drug release. These functional candidates offer excellent hydrophilicity by conjugating themselves to the sidewall of the CNT through chemical bond and they are also considered as a favourable material typically used to reduce water surface tension^29^. In a recent investigation, Lopez and co-workers^30^ reported that the surface-modified CNT is unique in their ability to diffuse across the biological membranes such as lipid bilayer, which will lead to the enhancement of their uptake into mammalian cells^31^. Furthermore, several studies have indicated that the water-soluble functionalised CNT exhibits less cytotoxicity and oxidative stress^32^, and can even be excreted or degraded inside the human body compared to pristine CNT^33,34^.

Herein, a sustained drug delivery system was developed based on (i) surfactant-based polymers: polysorbate 20 or polysorbate 80 and (ii) biocompatible polymers: polyethylene glycol or chitosan, conjugated with MWCNT-COOH using silibinin (SB) as the model drug. SB, the major constituent of silymarin, is a potential anticancer drug treatment owing to its capability of exerting a variety of antiproliferative and anticarcinogenic effects in different cancer cell lines^35^. Despite such advantages, this flavonoid is a hydrophobic drug with poor water solubility of fewer than 50 μg/mL and this has limited its advancement in drug formulation development to achieve bioavailability. Hence, conjugating SB to the hydrophobic portions of CNT through hydrophobic-hydrophobic interaction will greatly enhance the drug loading efficiency of SB.

Polyoxyethylene sorbitan esters are non-ionic surfactant classically employed as a dispersing agent and emulsifier in pharmaceutical formulations due to its non-toxicity, good biocompatibility and excellent stabilising characteristic for proteins^36,37^. These surfactant agents are generally categorised as polysorbate (e.g. polysorbate 20, 40, 60 and 80). They share a common core structure of sorbitan etherified with polyoxyethylene chains and then partially esterified with fatty acids, such as lauric, palmitic, stearic or oleic acid, for polysorbate 20, 40, 60 and 80 respectively. The role of these amphiphilic molecules is to reduce protein surface tension and to protect protein from denaturation as well as aggregation at hydrophobic surfaces.

Chitosan (CHI), as a natural polysaccharide with positive charge in aqueous solution, has received a substantial amount of attention owing to its excellent biocompatibility, high biodegradability, low toxicity, anti-infection activity, abundant availability and good mucoadhesion properties. All these merits express by CHI have led to a broad range of potential biomedical applications, particularly in targeted drug delivery and regenerative medicine engineering^38,39^. Polyethylene glycol (PEG), on the other hand, can be a useful hydrophilic polymer for the surface coating of nanoparticles that resists uptake by the reticuloendothelial system^40^. This is because nanoparticles interact immensely with the endothelium cells from the blood vessels, hence they are rapidly degraded and removed from the blood circulation mostly by the mononuclear phagocyte system. With the utilisation of PEG combined with the nanoparticles, this will lead to an extension of drug circulation time and potentially improve the biodistribution of drug delivery system in vivo. Furthermore, PEG is widely used in nanomedicine and pharmaceutical research due to its classification as Generally Regarded as Safe (GRS) in humans by the US Food and Drug Administration.

Our group has previously reported the development of a pH-dependent, sustained-release of SB from CNT-based drug delivery system^41^. Its promising results inspired us to design a novel biocompatible MWCNT drug delivery system using water-soluble polymers with the aim of enhancing its solubility and versatility under physiological conditions. The MWSB nanocomposites developed in this work were modified with polysorbate 20 (denoted as MWSB-T20), polysorbate 80 (denoted as MWSB-T80), PEG (denoted as MWSB-PEG), CHI (denoted as MWSB-CHI) and their physico-chemical properties as well as biological features were thoroughly evaluated.

## Results and discussion

### Fourier transform infrared spectroscopy

**Figure 1:**
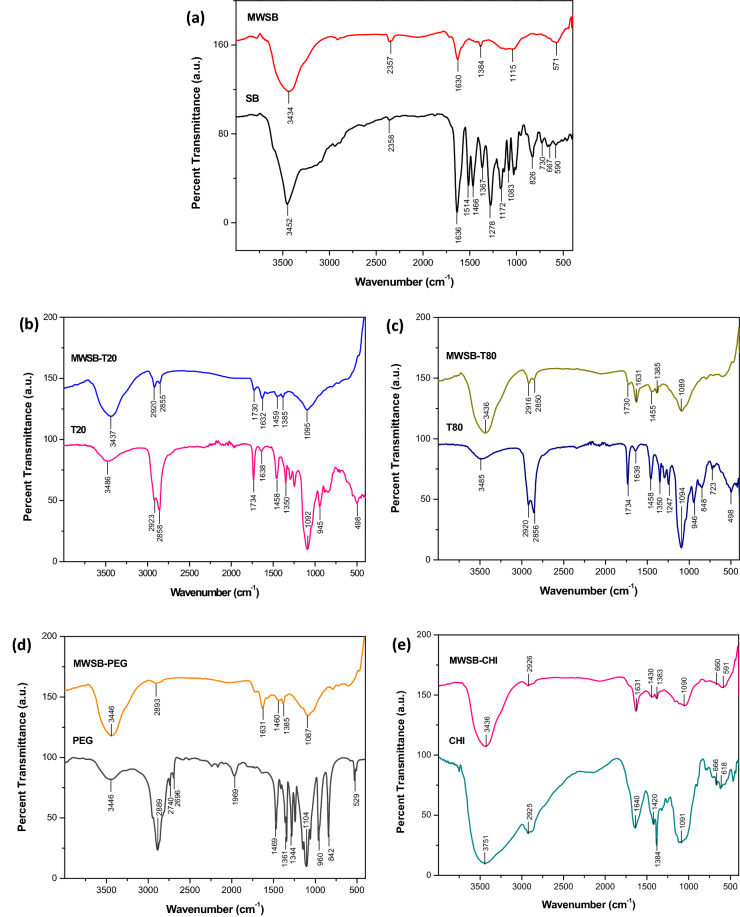
All samples were recorded over the range of 400–4000 cm^-1^ using the KBr disc method except for T20 and T80 by a direct deposition method. The FTIR spectra with characteristic absorption wavenumbers of the samples were shown as **(a)** SB and MWSB; **(b)** T20 and MWSB-T20; **(c)** T80 and MWSB-T80; **(d)** PEG and MWSB-PEG and **(e)** CHI and MWSB-CHI.

FTIR spectra of MWCNT-COOH, pure SB and SB-loaded MWCNT (Fig. 1a), along with their chemical structures had been extensively discussed in our previous report^41^. Therefore, in this paper the FTIR analysis is mainly focused on the interaction between hydrophilic polymers and SB-loaded MWCNT, which investigates the functional groups of four different types of biocompatible polymers used in the coating application.

The distinctive FTIR band feature of T20 observed in MWSB-T20 confirmed the successful coating of T20 onto the surface of MWSB nanocomposites (Fig. 1b). The absorption band of MWSB-T20 showed a strong and broad peak occurred at 3437 cm^−1^ corresponds to O–H stretching (characteristic absorption of MWSB) while the peaks centred at 1730 cm^−1^ and 1459 cm^−1^ can be attributed to C=O stretching of the ester groups and C–H bending in the methylene groups (characteristic absorptions of T20), respectively. Other FTIR bands like 2920 cm^−1^ and 2855 cm^−1^ are correlated to the asymmetric and symmetric C-H stretching vibrations of the methylene groups in T20. T80 is another type of polysorbate which has a similar chemical structure as T20 except that it exhibits a longer tail of fatty acid ester moiety. Therefore, the band adsorption of T80 as presented in Fig. 1c was closely related to T20 in Fig. 1b. Majority of the band positions observed in MWSB-T80 nanocomposites were identical to those of pure T80, suggesting a possible interaction between the nanocomposites and the polysorbate coating agent.

PEG is a primary alcohol that contains a hydroxyl group (O–H) that is attached to the hydroxyl carbon (C–OH) as a linkage. All the characteristic absorption bands of PEG were observed in the sample of MWSB-PEG with insignificant changes (Fig. 1d). They were typically found at 3446 cm^−1^ (O–H stretch), 2893 cm^−1^ (C–H stretch), 1631 cm^-1^ (C=C stretch), 1460 cm^−1^ (C–H bend) and 1385 cm^−1^ (in-plane O–H bend). It is also worth noting that the characteristic band found at 1087 cm^−1^ which is related to the repeating units (O-CH_2_-CH_2_) of PEG was not seen in the absorption band of the MWSB sample. These findings indicate that the PEG molecules interacted with MWSB nanocomposites through surface functionalisation.

Figure 1e shows the FTIR spectra of pure CHI and CHI-coated MWSB nanocomposites. CHI demonstrated principal absorption bands centred at 3751 cm^−1^ for O–H groups, 2925 cm^−1^ for stretching vibration of aliphatic C–H groups, 1640 cm^−1^ for stretching vibration of acetylated amino groups and 1420 cm^−1^ for bending vibration of –CH_2_ groups. The absorption band found at 1384 cm^−1^ can be credited to the primary amino moiety positioned at C_2_ of glucosamine^42^ while the strong, intense band occurred at 1091 cm^−1^ is related to C–O stretching vibration of ether. All of these characteristic bands were evident in the CHI-coated MWSB sample except the band of 2925 cm^−1^, 1420 cm^−1^ and 1091 cm^−1^, which were not detected in the case of MWSB. The disappearance of these bands can be deduced as the proof of a successful functionalisation reaction, possibly due to the electrostatic attraction between the two groups of ions with an opposite charge, such as carboxylate (–COO^−^) anions of the nanotubes and ammonium (–NH_3_^+^) cations of the CHI.

### Raman spectroscopy

**Figure 2:**
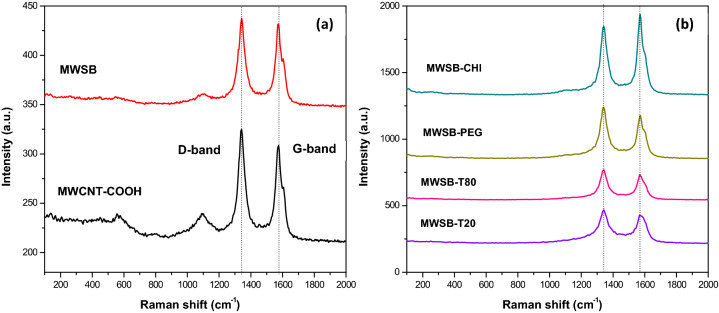
Raman spectra were acquired at the excitation wavelength of 532 nm. The samples were ultrasonicated for 10 min at room temperature and several drops from the suspension were placed on glass slides prior to the measurements. Raman detailed scans were carried out in the range between 100 and 2000 cm^−1^. **(a)** MWCNT-COOH, MWSB and **(b)** various types of biopolymeric MWSB nanocomposites.

To investigate the effect of the chemical interaction between the polymers and MWSB nanocomposites, Raman spectroscopy was used and the spectra were recorded in Fig. 2. The Raman spectroscopy is a non-destructive analytical tool (requires minimal sample preparation) widely used in chemistry to detect vibrational energy modes of a molecular system based upon the interaction of inelastic scattering light. Typically, this powerful tool is used to probe structural and chemical composition for different types of sp^2^ carbon nanomaterials and this includes CNT and graphene^43^. The most intense Raman features obtained from a CNT sample are the radial breathing mode (RBM), tangential mode (G-band) and disorder-induced mode (D-band) as well as other relatively small peaks. The RBM normally occurs between 120 cm^-1^ and 250 cm^-1^ in the Raman spectrum is related to the radial vibrations of the carbon atoms and it signifies the presence of SWCNT (single) or DWCNT (double). However, this low frequency mode is not detected in large diameter tubes like MWCNT because the intensity of the RBM feature is much weaker and is hardly noticeable.

Raman spectra displayed in Fig. 2a indicates that there were only two prominent peaks, namely the D-band and G-band appeared at 1342 cm^-1^ and 1575 cm^-1^ respectively, and they were generally present in all biopolymer-coated MWSB samples as seen in Fig. 2b. The D-band peaks are related to the breathing mode of the carbon hexagons that are activated by defects present in the carbonaceous nanomaterials whereas the G-band peaks are due to the first-order Raman-allowed *E*_2g_ phonon at the Brillouin zone centre. Thus, the combination of these two signature Raman peaks act as a good indicator to quantify the amount of structural disorder, in which a higher D to G ratio (normalised intensity ratio of I_D_/I_G_) indicates a higher defect density. Based on Table 1, the value of I_D_/I_G_ decreased by 0.029% after MWCNT-COOH was successfully loaded with SB. Thereafter, the defect density value showed a gradual increase in the range from 0.069% to 0.090% after MWSB was coated separately with surfactants and polymers. This indicates that the coated MWSB encountered minor destructive effects on the surface of MWCNT. However, this was not the case for MWSB-CHI in which the I_D_/I_G_ ratio decreased by a value of 0.129%. Analysis of Raman spectra evidenced that not all coating processes will eventually impart a higher defect density in the nanocomposites but indeed, it depends very much on the types of surfactants and polymers used in the application.

**Table 1:** The relative intensity of Raman peaks for all MWCNT samples.

Samples	D-band position (cm^-1^)	G-band position (cm^-1^)	I_D_/I_G_ intensity ratio
MWCNT-COOH	1342	1575	1.052
MWSB	1342	1575	1.021
MWSB-T20	1342	1567	1.091
MWSB-T80	1342	1567	1.099
MWSB-PEG	1342	1571	1.113
MWSB-CHI	1342	1571	0.889

Overall, Raman spectroscopy was found exceptionally useful in this case when analysing surfactants and polymers because they display no spectral features in the range from 1300 cm^-1^ to 1700 cm^-1^. For the analysis of biopolymeric MWSB samples, Raman scattering signals of the D-band and G-band intensity peaks were significantly weaker and slightly shifted toward lower frequencies compared to that of MWSB. The relative reduction of the characteristic peak intensity probably due to the formation of chemical interaction between the outer surfaces of MWSB and biopolymer molecules.

### Morphological observation

**Figure 3:**
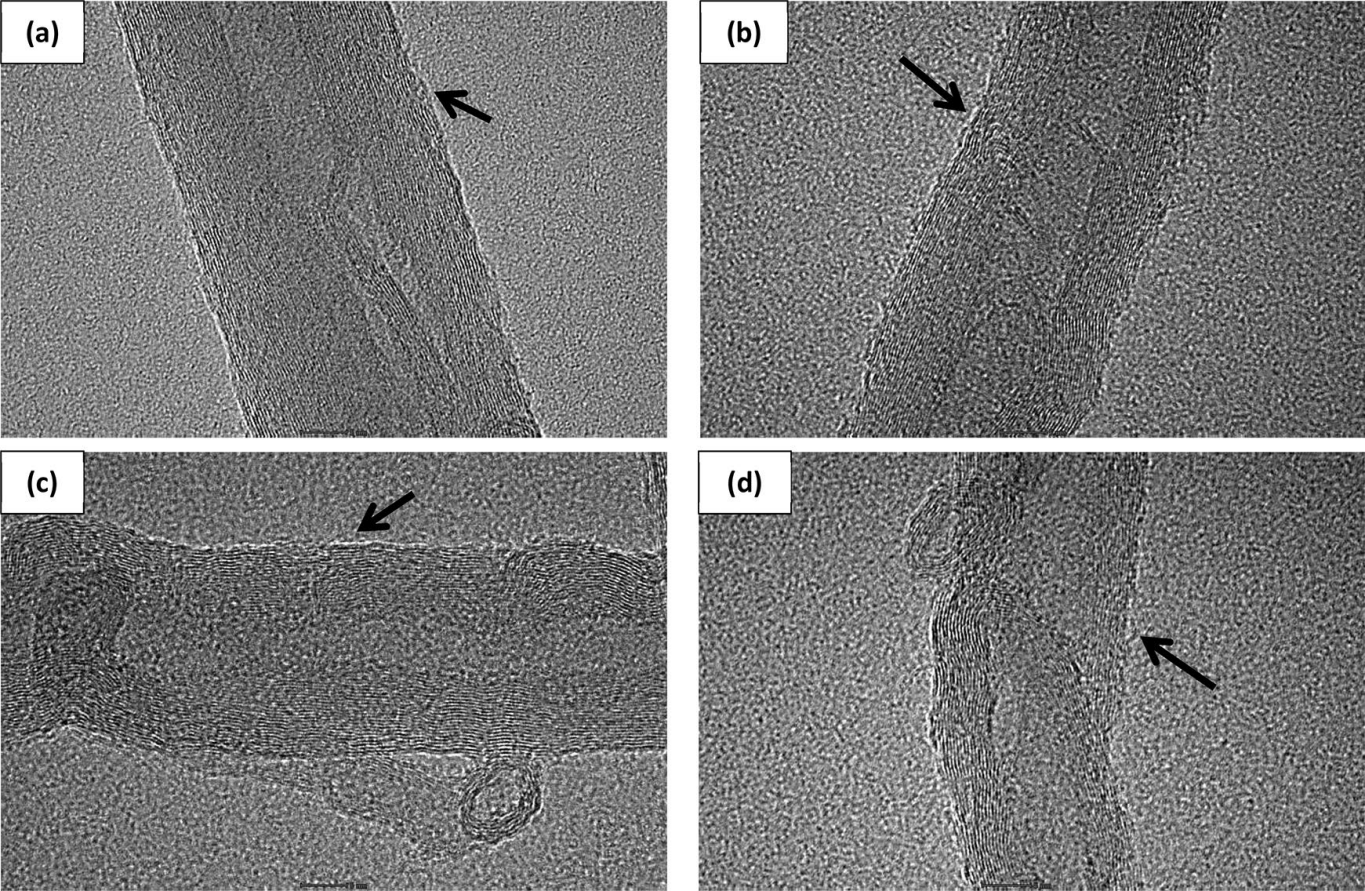
The samples were dispersed in ethanol by ultrasonication and dropped on the 300-mesh holey lacey carbon grids. Ethanol was then evaporated at room temperature and the cross-sectional HR-TEM images were captured and recorded as **(a)** MWSB-T20, **(b)** MWSB-T80, **(c)** MWSB-PEG and **(d)** MWSB-CHI. The arrow points to the thin layer of biopolymer coating deposited on the outer surface of nanotubes.

Figure 3 illustrates the HR-TEM images of the cross-sectional MWSB nanocomposites after coating with the respective biomaterials. The graphitic structure revealing multiple layers of the MWCNT was clearly visible under high-resolution mode. It was found that the surface morphology of the samples had changed with a thin layer of compound (~ 1 nm) wrapped on the outer surfaces of biopolymeric MWSB nanocomposites. This observation was consistent with the previous finding reported earlier by a team working on plasma-coated MWCNT^44^. According to the HR-TEM analysis, they observed a layer of thick amorphous plasma nano-coated on the MWCNT surfaces when compared to the uncoated MWCNT. There was also another group of scientists reported on the surface modification of MWCNT with octadecylamin molecules using HR-TEM technique^45^. They discovered that the surface morphology of the altered MWCNT became rougher due to the deposition of the modifier agent.

**Figure 4:**
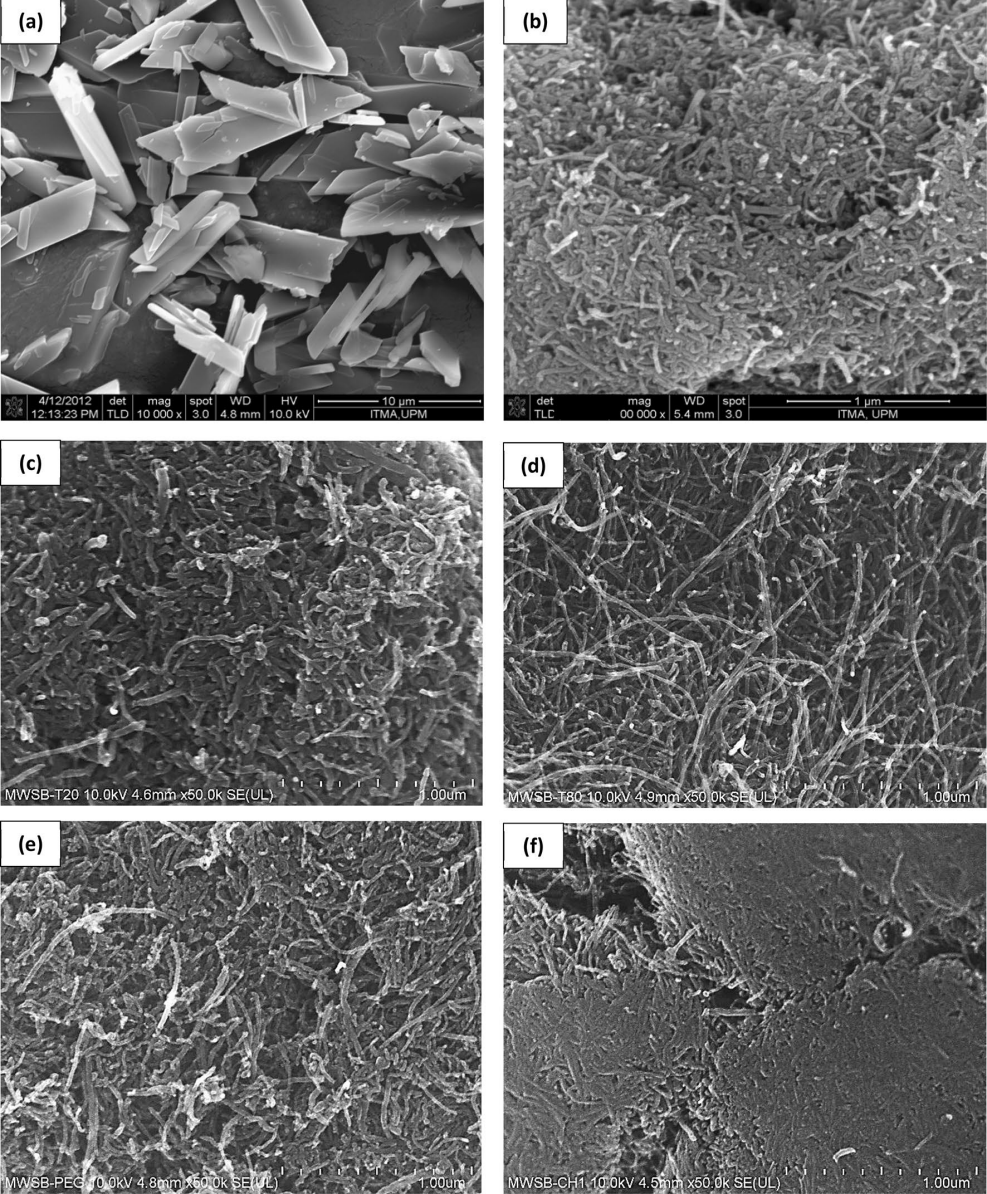
The samples were placed on an aluminium sample holder using double-sided carbon tape and the corresponding FESEM images were obtained at 10 kV. **(a)** SB at magnification ×10,000; **(b)** MWCNT-COOH at magnification ×100,000; **(c)** MWSB-T20 at magnification ×50,000; **(d)** MWSB-T80 at magnification ×50,000; **(e)** MWSB-PEG at magnification ×50,000 and **(f)** MWSB-CHI at magnification ×50,000.

Surface morphology of the SB-loaded nanocomposites plays a central role in the drug binding and release mechanisms. FESEM micrographs of the MWCNT-COOH, pure SB and the four different types of biopolymer-coated MWSB nanocomposites were displayed in Fig. 4. Generally, the morphology of the nanotubes (Fig. 4c–f) was observed to be heterogeneously-dispersed compared to the commercially-purchased MWCNT-COOH bundles (Fig. 4b). This could be possibly due to the consequence of a sample’s preparation using sonication process that utilises ultrasound energy to agitate and break down CNT aggregates. Additionally, the FESEM images also showed that the MWSB coated with T20, T80 and PEG displayed similar morphology except for MWSB-CHI which had quite smooth surfaces due to partially compact formation of the CHI polymer. This phenomenon could be triggered by the strong electrostatic dipole–dipole interaction resulting from the surface-charged of the CHI molecules and the carboxylated nanocomposites. Due to this reason, it caused a lower defect density in the MWSB-CHI sample, which was in good agreement with the Raman results. Besides, FESEM images also clearly revealed that all MWSB nanocomposites retained their micro-tubular shapes even after the sonication and coating process were applied. This indicates that the approach of sonication process and polymer coating were not destructive to the samples and thus, it will not induce a physical impact on the shapes of the nanotubes.

### Thermogravimetric analysis

**Figure 5:**
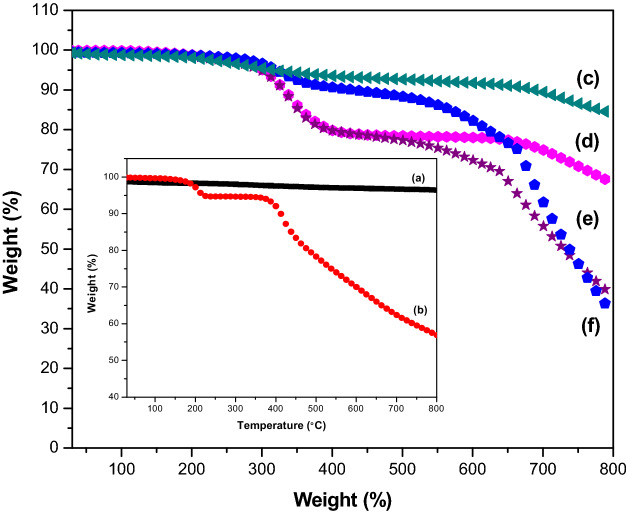
TGA curves were plotted by measuring the mass of samples during heating from ambient temperature up to 1000 °C with a heating rate of 10 °C/min under a nitrogen purge of 40 mL/min. **(a)** MWCNT-COOH, **(b)** pure SB, **(c)** MWSB-CHI, **(d)** MWSB-T20, **(e)** MWSB-PEG and **(f)** MWSB-T80.

Thermogravimetric method (TGA and DTG) is a technique widely used in the determination of surface functionalisation and thermal stability of materials based on weight loss or gained as a function of temperature and their thermal properties can easily plot into a simple thermogram. In general, a TGA thermogram is divided into few stages of decomposition: (i) the first stage is related to the release of water moisture; (ii) the subsequent stages are associated with the structural decomposition of polymers and finally combustion of the remaining material. Figure 5 displays the TGA curves and the weight loss of six samples, namely MWCNT-COOH, pure SB, MWSB-T20, MWSB-T80, MWSB-PEG and MWSB-CHI when plotted against temperature up to 800 °C.

In general, it was found that all four biopolymeric MWSB samples (Fig. 5c–f) demonstrated a different thermal property when compared to MWCNT-COOH (Fig. 5a) and pure SB (Fig. 5b) alone, suggesting crosslink reaction occurred which subsequently changed the TGA curves of the coated samples. The addition of MWCNT-COOH as drug delivery vehicle significantly increased the effective thermal conductivity of SB from 200 °C to approximately 300 °C as it shifted towards the higher temperature. This can be explained by the hydrophobic forces of SB molecules being attracted to the hydrophobic sites of MWCNT-COOH through physico-chemical adsorption.

As seen in Fig. 5a, MWCNT-COOH demonstrated relatively higher thermal stability due to its excellent temperature resistance when compared to pure SB in Fig. 5b, generating a total weight loss of about 19.12%. The weight loss of the MWCNT-COOH mainly contributed by the decomposition of free carboxyl and hydroxyl groups presented on the surface of the nanotubes. Furthermore, the carboxyl groups grafted on the surfaces of MWCNT during the oxidation process reacted to form a network of hexagonal hydrogen bonds and subsequently improved the degradation’s activation energy of MWCNT^46^. SB, on the other hand, is a natural antioxidant that has a broad range of thermal stability and due to this reason, it could be divided into three different endothermal stages as presented in Fig. 5b. The first stage of decomposition normally corresponds to the water crystallisation recorded between 50–120 °C whereas the second and third stages of thermal decomposition only began at 252 °C with a total weight loss of 57.33%. Based on Fig. 5c–f, the TGA curves of the four biopolymer-coated MWSB nanocomposites showed different thermal profiles due to different thermal properties possessed by the polymer themselves. Among the four samples, CHI-coated MWSB demonstrated excellent thermal stability, followed by PEG-coated MWSB, T80-coated MWSB and lastly T20-coated MWSB, with an overall weight loss of about 31.05%, 57.32%, 37.24% and 56.22% respectively.

**Figure 6:**
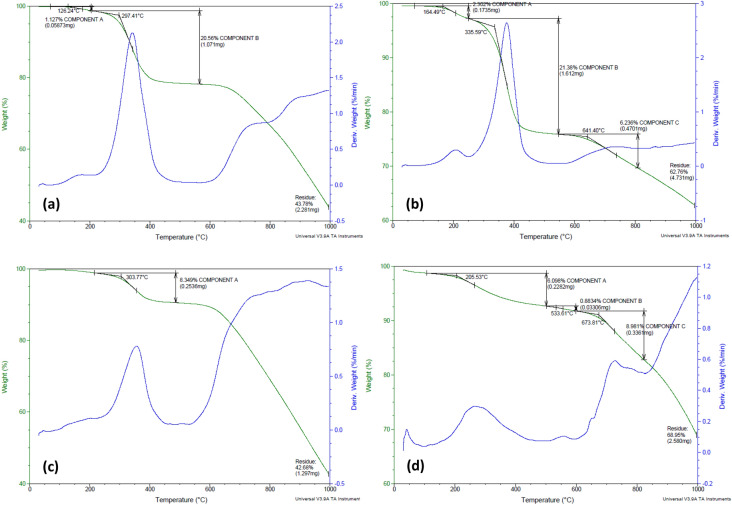
The TGA-DTG thermogram profiles of **(a)** MWSB-T20, **(b)** MWSB-T80, **(c)** MWSB-PEG and **(d)** MWSB-CHI.

To study the thermal profile of the four biopolymeric MWSB nanocomposites in more details, the TGA curves of the samples were converted into a derivative thermogravimetry (DTG) curves as shown in Fig. 6, which determine the rate of sample weight loss with respect to time. Since T20 and T80 are derived from the same polysorbate family and thus, they showed almost comparable thermal characteristic in the temperature range from 100 to 500 °C (Fig. 6a,b) with a total weight loss of approximately 56.22% and 37.24%, respectively. A possible explanation underlying this differential effect of polysorbates is that, the molecular configuration of polysorbate 20 is more stabilised and highly structured as more bulk water is converted from the structured water molecules. Therefore, total weight loss observed was considerably higher as the decomposition temperature of MWSB-T20 increased^47^. However, polysorbate 80 was more thermally stable because it contains a mixture of multiple different polymer chain lengths. It displayed a weight loss of 27.62% in the second and third stages of thermal decomposition with its temperature increased up to 335.6 °C and 641.4 °C respectively as compared to polysorbate 20.

As shown in Fig. 6c, significant weight loss observed in the range of 200–600 °C in MWSB-PEG suggests the amino functional groups were thermally stable and they could take part in some chemical reactions with a temperature of 600 °C and above. A comparable observation also reported by a group of researchers on the drug release study for PEG-functionalised MWCNT incorporated with gelatin and CHI^48^. They discovered that the thermal decomposition of carboxylated and PEG-grafted MWCNT occurred between 90 and 600 °C with MWCNT-PEG resulted in the highest weight loss overall. For the case of CHI-coated MWSB nanocomposites, the sample was thermally stable up to 200 °C, where the weight loss is commonly attributed to water desorption (Fig. 6d). Subsequently, the thermogram curve experienced a small slope at about 500 °C which could be due to the release of volatile compounds consist of NH_3_, CO, CO_2_, and CH_3_COOH during the decomposition of CHI as evidenced in the FTIR analysis. After this temperature, another weight loss took place at 674 °C and caused a modest weight loss of about 9%. This is the second stage of decomposition that was characterised by the production of CH_4_ and the formation of the graphitic structure as a result of pyrolysis reaction when CHI was almost completely combusted^49^.

Among the coated MWSB samples, MWSB-CHI nanocomposites emerged as the lowest of the overall weight loss which was mainly due to the decomposition of CHI. This is correlated to the degree of deacetylation and the low molecular weight of CHI used in the study. A similar observation was published by a team on a study of thermal degradation and water adsorption capacity in which, they compared the thermal properties of commercial chitosan (degree of deacetylation = 75% to 85%, medium molecular weight) and medical grade chitosan (degree of deacetylation > 93%)^50^. The scientists found out that the commercial chitosan showed a slightly higher weight loss of about 67% as compared to medical grade chitosan where the weight loss observed was much lower (51 wt%). They attributed this finding to the amount of acetic acid released in the pyrolysis of CHI during thermal decomposition since the volatile compound is closely related to the degree of deacetylation. In addition to that, another feasible reason could be contributed by the formation of a strong hydrophobic bond between the electrostatic interaction of ammonium cations of CHI and the carboxylate anions attached to MWCNT-COOH (as a product of acid treatment) which caused the nanotubes to be easily rearranged themselves within the CHI polymer chain.

Based on the TGA-DTG curves, it could be further deduced that the incorporation of coating agent significantly enhanced the thermal stability of all four biopolymeric MWSB samples in comparison to the uncoated one. MWCNT-COOH is well known for its high thermal stability as it is capable of forming many cores of heat conduction which generates the crosslinking points between the surface of the nanotubes and the polymer matrix. The thermal stabilisation effect of MWCNT-COOH on the nanocomposites could be justified as, the nanotubes and their aggregates form a barrier to prevent the diffusion of the degraded materials from the polymer matrix enter the gas phase. Moreover, the carboxyl groups on the surfaces of MWCNT could proliferate the interfacial bonding between the nanotubes and the polymer matrix, which further improves the degradation’s activation energy.

### SB loading and release studies

PBS is a water-based salt solution ubiquitously used in many biological applications, such as transporting of cells or tissues, rinsing of cells before dissociation and diluting substances as it helps to maintain a constant pH (~ 7.4). It can be easily prepared from the combination of four different types of salt solutions, namely NaCl with Na_2_HPO_4_ or KCl with KH_2_PO_4_ in the presence of distilled water. It is popularly used in drug release study by pharmaceutical scientists as it closely mimics the pH, osmolarity and ion concentrations of the human body. Therefore in this work, the release pattern of SB was investigated in PBS (~ pH 7.4) at ± 37 °C which represents simulated body fluid and the amount of drug released was determined by UV–vis spectrophotometer on a real-time drug release mode.

**Figure 7:**
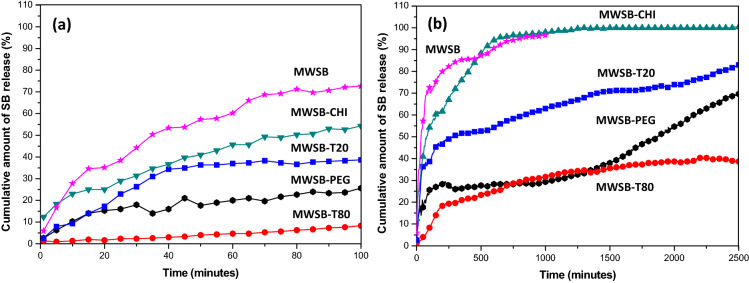
Controlled-release profiles of the samples in PBS (pH 7.4) were characterized at λ_max_ = 288 nm (characteristic wavelength of SB) in a quartz cell with a 1.0 cm path length. The SB sustained-release curves were plotted from uncoated and coated MWSB samples in PBS at pH value of 7.4 and ± 37 °C.

Figure 7a shows the first 100 min of the initial release of SB from five different formulations whereas the total release equilibrium up to 2500 min was recorded in Fig. 7b. The drug release results demonstrated that the uncoated MWSB and coated MWSB exhibited a similar release profile during the drug dissolution test, suggesting that the incorporation of polymers in the nanocomposites did not alter the release pattern of SB. Based on Fig. 7a, the initial release of SB from uncoated MWSB was found to be about 72.7% within 100 min, indicating a burst release effect. Upon addition with the surfactants and polymers, i.e. T20, T80, PEG and CHI, the burst release effect was reduced substantially to 25.7%, 8.3%, 38.7% and 54.3% respectively. This is because the polymers act as a shielding effect between the nanotubes and the surrounding aqueous medium which significantly delay the diffusion of SB molecules into the buffer solution through the polymer matrix. In all cases, SB was released at a steady, slow rate due to its limited water solubility at the beginning of the study. After about 100 min, a substantial amount of drug was released from the nanocomposite formulations possibly caused by the swelling and disintegration behaviour of the polymer themselves. After 1000 min, the released of SB from uncoated MWSB was nearly 96.6% whereas the coated MWSB presented a much slower and sustained-release behaviour. During the following days, the amount of released SB from coated nanocomposites could still be observed even after 2500 min, except for MWSB-CHI which had achieved total release equilibrium of about 100% at 1500 min.

In comparison to uncoated MWSB, the released of SB from MWSB-T20, MWSB-T80, MWSB-PEG and MWSB-CHI represent a better version of sustained drug release behaviour. Among the four coated MWSB nanocomposites, it was noted that the MWSB-CHI exhibited the highest release rate of SB molecules in the isotonic buffer solution overall. This could be attributed to a much lower amount of the polymer content in the nanocomposite, which was about 8.01% as analysed by the TGA experiment earlier. Besides, the CHI coating could have been dissolved completely at 1500 min thereby releasing 100% of SB into the PBS solution, resulting in the plateau saturation curve of MWSB-CHI. Due to the high water uptake property of CHI, it became unstable in PBS as the salt ions were formed between the carboxylate ions of the oxidised nanotubes and ammonium ions of the CHI polymer chains and eventually, they underwent complete disintegration. As for the remaining three nanocomposites, the cumulative amount of SB released from MWSB-T20 was found to be around 69.7%, MWSB-PEG was nearly 83.0% and MWSB-T80 was only about 38.7% within 2500 min at pH 7.4.

The difference observed in the release profile of MWSB-T20 and MWSB-T80 despite having a similar chemical structure is that, the amount of SB released from MWSB-T20 was significantly faster than MWSB-T80 as seen in Fig. 7b. This observation may be explained by the ionisation of the polymeric chains due to partial hydrolysis of ester functional groups that result in producing more charged carboxylate ions. The nanocomposites might have experienced different degree of swelling due to the repulsive forces between the ionised carboxylate ions and the salt ions in the release medium. Subsequently, the conjugated SB molecules were easily released through the polymer layer and then diffused into the external medium due to weak hydrophobic interaction forces between the drug and the nanotubes. As for the MWSB-PEG nanocomposites, they contain the functional groups of –CH_2_OH and –CH_2_CH_2_O– attached to their outer surfaces as a product of PEG treatment which substantially speed up the solubilisation of hydrophobic drug, SB. Thus, the drug was instantaneously diffused from the PEG shell at the beginning stage followed by a slow and sustained-released into the PBS medium by capillary effect with the erosion of the polymer.

The nanocomposite formulations with the incorporation of polymers synthesised in this work showed a biphasic release pattern with an initial burst effect within the first 100 min followed by a constant rate of sustained-release over a long period. This enhanced feature demonstrated by the nanocomposites was comparable to some of the reported work as presented in Table 2. Hence, it could be postulated that the coated MWSB nanocomposites with SB loading capacity of about 37% are capable of prolonging the circulation time of the drug in the blood stream for more than 2500 min under physiological conditions. Furthermore, the in vitro release experiment indicates that the drug release behaviour can be tailor-made by cautiously selecting the desired biopolymer as the coating agent to suit various biomedical purposes, such as slow and sustained-release for anticancer therapy or initial burst effect for anaesthetic medicine to promote immediate pain relief. Hence, this essential parameter can be used effectively as a method for controlling the release rate of drug molecules from the delivery vehicle like MWCNT.

**Table 2:** Comparison of in vitro release data of hydrophobic drug loaded onto MWCNT as a drug delivery vehicle to improve solubility. All tests were conducted in PBS (pH 6.8–7.4) as the dissolution media for the drug release study.

Samples	Drug loaded	Maximum drug released (%)	Overall time released (min)	Ref
MWCNT-COOH/gelatin-CHI MWCNT-PEG/gelatin-CHI	Ciprofloxacin lactate (10 μg)	97.5 98.1	90	^48^
MWCNT	Dipyridamole (50 wt%)	40.0	60	^51^
MWCNT-COOH	Griseofulvin Sulfamethoxazole (N/A)	80.0 80.0	120 52.5	^52^
MWCNT-PEG	Methotrexate (56.5 wt%)	44.2	1680	^53^
MWCNT-COOH	6 mercaptopurine (65 wt%)	96.1	1200	^54^
MWCNT-glucose/starch	Zolpidem (75 wt%)	64.0	4320	^55^

### Release kinetics mechanism

The drug release kinetics is the most critical and pre-requisite feature in the development and characterisation of an effective drug delivery system for biomedical applications. With this in mind, the in vitro release profiles of the uncoated and coated MWSB were fitted to five kinetic equations (including the zeroth-order, pseudo-first order, pseudo-second order, Higuchi and Korsmeyer-Peppas) and the results were summarised in Table 3. When the highest linearity of the correlation coefficients (R^2^) for SB released from different MWSB nanocomposites were compared, it was found to follow the pseudo-second order with R^2^ values ranging from 0.9909 to 0.9980. This implies that the drug release mechanism is a second order reaction governed by the rate-controlling step which depends mainly on diffusion-limited processes through chemisorption reaction^56,57^.

**Table 3:** The correlation coefficient (R^2^) obtained by fitting the SB release data collected from various MWSB formulations.

Sample	Saturation release (%)	R^2^				
		Zeroth	Pseudo-first	Pseudo-second	Higuchi	Korsmeyer-Peppas
MWSB	97	0.6284	0.9491	0.9980	0.7883	0.8158
MWSB-T20	31	0.3859	0.4253	0.9972	0.5342	0.6764
MWSB-T80	41	0.7406	0.8508	0.9929	0.8887	0.8866
MWSB-PEG	74	0.8394	0.9436	0.9909	0.9342	0.8772
MWSB-CHI	99	0.8004	0.9771	0.9939	0.9295	0.9790

### Cytotoxicity assay

Previously we have reported cytotoxicty action of an uncoated MWSB formulation on human cancer cell line (HepG2 cells and A549 cells) in vitro^41^*.* The MTT results indicated that MWSB formulation retained its anticancer activity and subsequently demonstrated enhanced cytotoxicity activity in human cancer cells compared to free SB at lower dosages. This has motivated us to further improve on the biocompatibility of the MWSB formulation with the use of water-soluble biomaterials like polysorbates, PEG and CHI. Eventhough these hydrophilic polymers are widely explored in biomedical science related applications, their biological effects of their surface compatibility are still not extensively explored^58^. Therefore, it is mandatory to evaluate the cytocompatibiliy of the MWSB formulations using MTT assay as a preliminary in vitro study.

**Figure 8:**
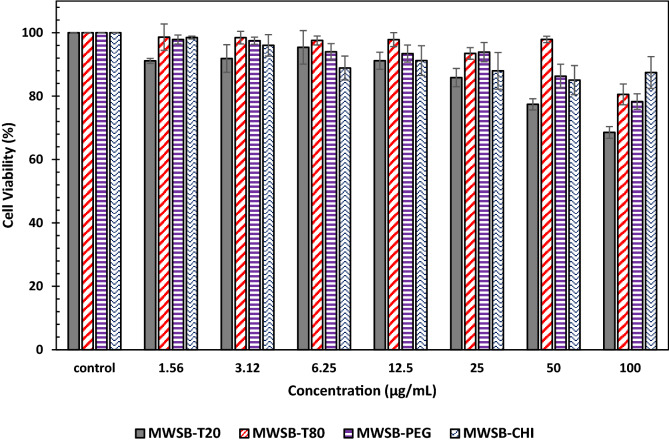
3T3 viable cells exposed to various concentrations of biopolymer-coated MWSB nanocomposites for exposure up to 72 h. Control wells did not contain any of the nanocomposites but 3T3 cells only. Error bars represent the standard deviation (*n* = 3). Statistical difference between different groups of concentrations and within the group of concentration (p < 0.05).

In view of this, the cytotoxicity activity of the coated MWSB nanocomposites was evaluated on a 3T3 cell line for an incubation period of 72 h. 3T3 mice fibroblasts were chosen in the current investigation is because of their wide range of biomedical applications in cell culture for several decades^59^, including toxicity tests and antioxidant action of biomaterials in drug design research^60–62^. To determine the cytotoxicity action of the samples, the cells were exposed to varying concentrations of MWSB-T20, MWSB-T80, MWSB-PEG and MWSB-CHI (1.56–100 μg/mL) which was previously solubilised in PBS. The cell metabolic activity was then evaluated in a concentration-dependent manner according to the standard MTT assay protocol^63^ and the results were depicted in Fig. 8.

Based on a previous report^64^, MWCNT-COOH is known to induce cytotoxicity at the highest concentration of 50 μg/mL in a 3T3 cell line, indicating that a safe dose of fewer than 40 μg/mL of MWCNT-COOH could only be utilised in drug delivery^65^. However, the present investigation revealed that all concentrations were observed to display maximum cell viability (between 69 and 98%) which was comparable to untreated cells. The mortality rate of the 3T3 cells was found to be at the very minimal extent in MWSB-T20 nanocomposites, reaching to about 68.6% of viable cells at the highest concentration of 100 μg/mL. The plausible reason for no cytotoxicity activity in all four coated MWSB nanocomposites is due to their reported biocompatibility and biodegradability of polysorbates, PEG and CHI, rendering them potentially safe drug carriers for nanomedicine. Our finding herein suggests that the surface coating of nanotubes with hydrophilic polymers significantly enhanced the biocompatibility and solubility of the MWCNT-COOH since CNT has been a major concern for their use in biomedical applications. For future development, it is essentially important to assess the pharmacokinetic profile of the nanocomposites, cell cellular uptake and clearance from the human body which remain the most challenging task to achieve in the field of nanomedicine.

## Methods

### Chemicals

The detailed parameters for the purchased short carboxylated MWCNT (MWCNT-COOH) were as follows: outside diameter 20–30 nm; length 0.5–2.0 μm; purity > 95 wt%; COOH functional content 1 – 2 wt%; ash < 1.5 wt%. SB (> 98% purity with the molecular formula C_25_H_22_O_10_), CHI (deacetylation degree = 75–85%, low molecular weight) and PEG (average molecular weight 300) were used in the experiment. Ethanol (≥ 99.8%) was used as a solvent for SB and aqueous acetic acid solution (99.8% purity) was used as a solvent for CHI.

### Preparation of SB-loaded MWCNT nanocomposites

The procedure used to synthesise SB-loaded MWCNT was described in our previous work using non-covalent method without employing a cross-linker agent^41^. In brief, 400 mg of MWCNT-COOH was added to SB solution at the optimised drug concentration of 0.05 mg/mL and ultrasonic irradiated for approximately 1 h to mechanically separate the nanotubes. After that, the suspension was reacted under constant magnetic stirring for 20 h at room temperature and the pH was slowly adjusted to 4 for optimum drug loading. The mixture was then filtered, centrifuged, washed with ethanol and deionised water simultaneously for three times and eventually dried in an oven at 60 °C overnight. The resulting product (MWSB) was grinded homogenously and stored in a sample bottle at room temperature for further use. The supernatant residue containing unbound SB was collected for the quantification of drug loading efficiency of MWCNT-COOH and the result was calculated according to the following formula:

1 $$Drug \,  loading\,  efficiency \left( \% \right) = \frac{{\left( {W_{initial\,  SB} {-} W_{unbound\,  SB} } \right)}}{{W_{initial\,  SB} }} \times 100\% ,$$

where W is the weight in mg, W_initial SB_ and W_unbound SB_ are the initial amount of SB and the amount of unbound SB in the supernatant residue, respectively.

### Preparation of non-ionic surfactant-coated MWSB nanocomposites

T20-coated MWSB nanocomposites were prepared according to literature with some minor modifications^66^. Approximately 100 mg of prepared MWSB was added to T20 (1% v/v) dissolved solution and continuously stirred for 24 h at room temperature. The precipitate was then collected, centrifuged and washed with deionised water three times to remove excessive T20 which was not participated in the coating process, and finally dried in an oven to yield MWSB-T20 nanocomposites. The surfactant-coated MWSB nanocomposites with T80 were prepared similarly and the final product was designated as MWSB-T80.

### Preparation of biopolymer-coated MWSB nanocomposites

To synthesise MWSB-PEG nanocomposites, 100 mg of MWSB was mixed with 1% PEG dissolved into 100 mL of deionised water. The mixture was magnetically stirred for 24 h and the resulting black precipitates were then collected, centrifuged and rinsed with deionised water three times. The final product was obtained after drying completely in an oven overnight. MWSB-CHI nanocomposites were prepared using similar method, except that CHI (0.5% v/v) was used as the coating agent for MWSB.

### Quantification of polymer content

As an effort to predict the wt% of the coating agents used in the preparation of MWSB nanocomposites, thermogravimetric analysis was performed on blank MWCNT-COOH coated with the respective surfactants and polymers. A set of four samples denoted as MWCNT-T20, MWCNT-T80, MWCNT-PEG and MWCNT-CHI were prepared under the same conditions as described above. Their estimated polymer content together with MWCNT-COOH as the reference, was summarised in Table 4.

**Table 4:** TGA analysis of the blank coated MWCNT samples.

Samples	Residue (wt%)	Estimated polymer content (wt%)
MWCNT-COOH	80.88	–
MWCNT-T20	61.92	18.96
MWCNT-T80	21.66	59.18
MWCNT-PEG	71.75	9.13
MWCNT-CHI	72.87	8.01

### Quantification of drug loading

In order to measure the loading efficiency of SB onto MWCNT-COOH, the supernatant residue collected in the preparation stage earlier was used in this analysis. The SB solution used for drug loading was also analysed as a basis of comparison to calculate the loading efficiency using UV–vis spectrophotometer. The signals generated from samples were correlated to a standard calibration curve achieved under the same condition and the drug loading efficiency was estimated to be about 37%. This value of SB loading efficiency is essentially higher than the encapsulation efficiency of CHI nanoparticles conjugated with SB^67^ which was only around 25%.

### In vitro drug release studies

The sample (MWSB-T20, MWSB-T80, MWSB-PEG or MWSB-CHI) was immersed in 3 mL of pH 7.4 phosphate-buffered saline (PBS) under sink condition to simulate blood physiological environment. The released amount of drug was measured by UV–vis spectrophotometer at 288 nm and the experiment was then terminated upon reaching saturation. To study the release kinetics of SB from biopolymeric MWSB nanocomposites, the drug release profiles were plotted and fitted into five mathematical models namely the zeroth-order, pseudo-first order, pseudo-second order, Higuchi and Korsmeyer-Peppas equation^68^.

### Cell line and cell culture

Normal and healthy mouse embryonic fibroblasts 3T3 cells acquired from ATCC were utilised for in vitro cytotoxicity assay. The cells were maintained as single layers in RPMI supplemented with 10% fetal bovine serum and 1% penicillin–streptomycin. The cells were then grown at 37 °C in a humidified atmosphere containing 5% CO_2_. When confluent (approximately 80%), the cell line was transferred and sub-cultured in a new culture flask for seeding and treatment purposes using 0.25% trypsin–EDTA solution. After that, stock solutions containing the biopolymeric MWSB nanocomposites were freshly prepared in PBS solution and diluted serially to the desired concentrations (0 μg/mL, 1.56 μg/mL, 3.12 μg/mL, 6.25 μg/mL, 12.5 μg/mL, 25 μg/mL, 50 μg/mL and 100 μg/mL). The cells cultured without nanocomposites were used as control group (cells in media only).

### Cell viability assay

Biocompatibility study of the biopolymeric MWSB nanocomposites was tested by MTT reagent [3-(4,5-dimethylthiazol-2-yl)-2,5-diphenyltetrazolium bromide]. 3T3 cells were seeded (1 × 10^5^ cells/well) in 96-well microplate and incubated at 37 °C (5% CO_2_ and 95% air) for 24 h to allow cell attachment. Subsequently, the cells were exposed to different concentrations for 72 h. After that, cells were incubated with 20 μL MTT reagent at 5 mg/mL in PBS for 3 h at 37 °C. Following incubation, excessive MTT was discarded and 150 μL of dimethylsulfoxide was added to dissolve the formazan dye formed in the assay. Finally, the cell viability can be observed at 570 nm with 630 nm as a reference wavelength, using EL 800X microplate reader. The absorbance value is directly proportional to the cell survival rate. It can be calculated with the formula:

2 $${\text{Cell viability}} = \frac{{\left( {A_{test} {-}{ }A_{blank} } \right)}}{{\left( {A_{control} { }{-}{ }A_{blank} } \right){ }}} \times 100\% ,$$

where *A*_test_ is the absorbance of cells exposed to biopolymer-coated MWSB nanocomposites, *A*_control_ is the absorbance of untreated cells and *A*_blank_ is the absorbance from empty wells. Mean values of absorbance were determined and all experiments were carried out in triplicate independently.

### Statistical analysis

All data were reported as mean ± standard deviation of triplicate (*n* = 3) for MTT assay and analysed by one-way ANOVA. Statistical analysis was performed using a 95% confidence interval (*p* < 0.05) with IBM Statistical Package for Social Sciences Statistics version 25.0 software.

## Data Availability

The research data and materials supporting this publication are available from the corresponding authors upon reasonable request.
